# Defective Leukocyte Adhesion and Chemotaxis Contributes to Combined Immunodeficiency in Humans with Autosomal Recessive MST1 Deficiency

**DOI:** 10.1007/s10875-016-0232-2

**Published:** 2016-01-22

**Authors:** Tarana Singh Dang, Joseph DP Willet, Helen R Griffin, Neil V Morgan, Graeme O’Boyle, Peter D Arkwright, Stephen M Hughes, Mario Abinun, Louise J Tee, Dawn Barge, Karin R Engelhardt, Michael Jackson, Andrew J Cant, Eamonn R Maher, Mauro Santibanez Koref, Louise N Reynard, Simi Ali, Sophie Hambleton

**Affiliations:** Institute for Cellular Medicine, Newcastle University, Newcastle upon Tyne, NE2 4HH UK; Institute of Genetic Medicine, Newcastle University, Newcastle upon Tyne, UK; School of Clinical and Experimental Medicine, University of Birmingham, Birmingham, UK; Department of Paediatric Allergy & Immunology, Royal Manchester Children’s Hospital, University of Manchester, Manchester, UK; Great North Children’s Hospital, Newcastle upon Tyne Hospitals NHS Foundation Trust, Newcastle upon Tyne, UK; Blood Sciences Flow Cytometry Laboratory, Newcastle upon Tyne Hospitals NHS Foundation Trust, Newcastle upon Tyne, UK; Department of Medical Genetics, University of Cambridge, Cambridge, UK

**Keywords:** Combined immunodeficiency, MST1, STK4, lymphocyte adhesion, chemotaxis, CD4 lymphopenia

## Abstract

**Purpose:**

To investigate the clinical and functional aspects of MST1 (*STK4*) deficiency in a profoundly CD4-lymphopenic kindred with a novel homozygous nonsense mutation in *STK4.* Although recent studies have described the cellular effects of murine Mst1 deficiency, the phenotype of MST1-deficient human lymphocytes has yet to be fully explored. Patient lymphocytes were therefore investigated in the context of current knowledge of murine Mst1 deficiency.

**Methods:**

Genetic etiology was identified by whole exome sequencing of genomic DNA from two siblings, combined with linkage analysis in the wider family. MST1 protein expression was assessed by immunoblotting. The ability of patient lymphocytes to adhere to ICAM-1 under flow conditions was measured, and transwell assays were used to assess chemotaxis. Chemokine receptor expression was examined by flow cytometry and receptor signalling by immunoblotting.

**Results:**

A homozygous nonsense mutation in *STK4* (c.442C > T, p.Arg148Stop) was found in the patients, leading to a lack of MST1 protein expression. Patient leukocytes exhibited deficient chemotaxis after stimulation with CXCL11, despite preserved expression of CXCR3. Patient lymphocytes were also unable to bind effectively to immobilised ICAM-1 under flow conditions, in keeping with a failure to develop high affinity binding.

**Conclusion:**

The observed abnormalities of adhesion and migration imply a profound trafficking defect among human MST1-deficient lymphocytes. By analogy with murine Mst1 deficiency and other defects of leucocyte trafficking, this is likely to contribute to immunodeficiency by impairing key aspects of T-cell development and function such as positive selection in the thymus, thymic egress and immune synapse formation in the periphery.

## Introduction

Primary immunodeficiencies (PIDs) are rare, inherited diseases characterised by the altered function or absence of immune cells. Recently, whole exome sequencing (WES) has emerged as a powerful tool for revealing the genetic etiology of certain PIDs. The benefits of this approach include the potential discovery of novel disease-associated genes, and the relatively efficient identification of novel variants within genes already known to be associated with PIDs. This work describes an example of the latter, specifically the discovery of a novel variant in the serine threonine kinase 4 (*STK4*) gene.

*STK4* encodes a 63kD protein known as MST1, a mammalian orthologue of the Drosophila Hpo protein, and a member of a family of kinases related to the yeast protein Ste20 (sterile 20) [1]. Soon after its discovery, MST1 was identified as having roles in regulation of apoptosis and proliferation [2, 3], with other Ste20-like kinases found to have roles in cytoskeletal rearrangement [4]. The role of MST1 in cell death is controversial, with both proapoptotic [5] and antiapoptotic [6] functions claimed, although MST1 deficiency has been associated with increased apoptosis in both mouse [7, 8] and human [9, 10]. MST1 is also a signalling intermediate in the process of ‘inside-out’ signalling in murine lymphocytes, converting signals initiated by chemokine stimulation into subsequent LFA-1 integrin activation and polarization [11, 12]. MST1 carries out this function by acting downstream of Rap1, a small GTPase that becomes active after ligation of chemokine receptors. Rap1 in turn binds RAPL, which binds to MST1. This complex then mediates the activation and polarization of the LFA-1 integrin, and localises with LFA-1 at the leading edge of the cell [12]. The surface redistribution of active LFA-1 is vital for transendothelial migration of lymphocytes, as well as for the formation of the immune synapse [13, 14].

Recently, two studies have identified MST1 deficiency as the cause of combined immunodeficiency (CID) in three different families with homozygous nonsense mutations in the *STK4* gene [9, 10]. This invariably resulted in profound CD4 lymphopenia and an accompanying phenotype of multiple bacterial and viral infections, and mucocutaneous candidiasis [15]. Here, we describe a further family with a biallelic *STK4* mutation and similar clinical features. Further analysis of patient cells revealed a defect in LFA-1-mediated adhesion and chemotaxis.

## Case Study

We studied three siblings with early childhood onset of CID characterized by CD4 lymphopenia and marked susceptibility to opportunistic and bacterial infection. The index patient (P2) was evaluated immunologically at 3 years of age following an episode of lobar pneumonia from which she had failed to recover fully. Her history was remarkable for a prior episode of viral laryngotracheobronchitis requiring intubation and ventilation, recurrent suppurative otitis media and eczema; she experienced severe primary herpetic gingivostomatitis, had ongoing chronic molluscum contagiosum and proved to be consistently EBV-viremic. Her elder sister P1 had a similar history of recurrent respiratory infection, molluscum and ongoing EBV-viremia when assessed in middle childhood. Both sisters also had documented cryptosporidiosis on more than one occasion and both ultimately developed EBV-associated lymphoproliferative disease, treated with Rituximab and steroids before stem cell transplantation. Their sister P3 was transplanted at a younger age because of a similar infection history (molluscum, history of severe primary HSV, recurrent herpes zoster) and the availability of a matched family donor. She developed florid cryptosporidial diarrhea on day 0 of transplant, most likely representing the recrudescence of chronic infection. After a stormy peri-transplant period with veno-occlusive disease, capillary leak and GVHD, she engrafted but had not immune reconstituted when she acquired primary CMV and went on to develop fatal immune dysregulation. P1 also succumbed to transplant-related complications in the form of a cerebrovascular accident shortly after conditioning, while P2 has had a good outcome from HSCT.

**Fig. 1:**
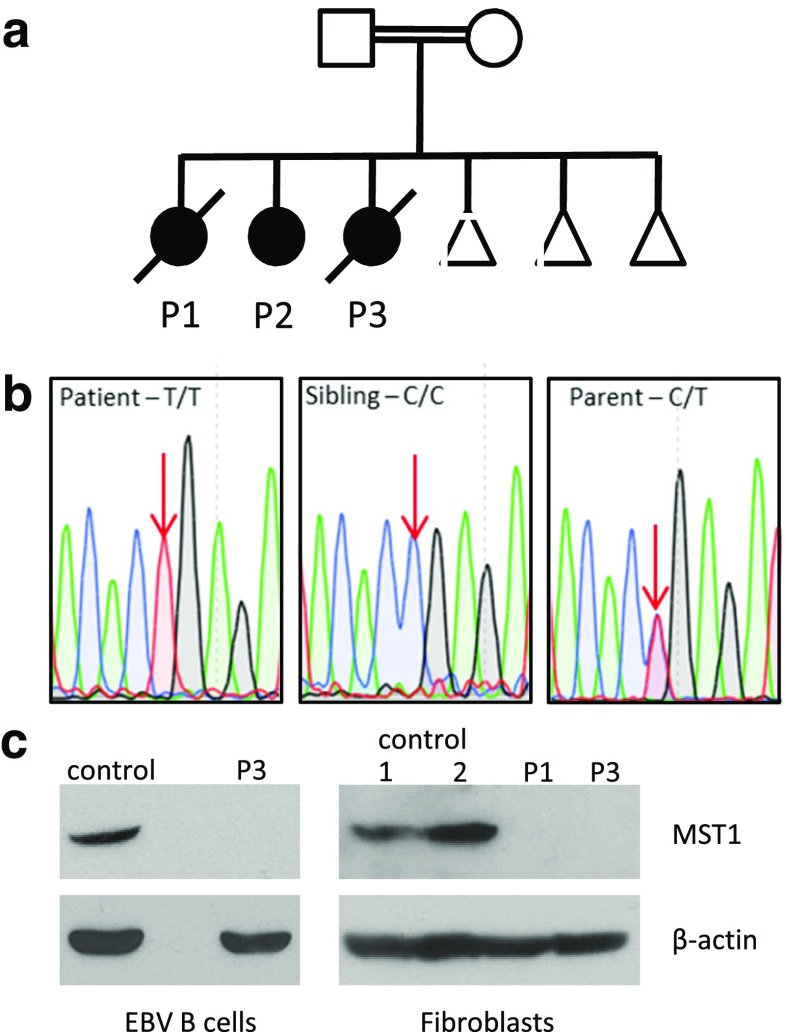
**a** Pedigree of affected family, demonstrating consanguinity (double horizontal lines). Males and females are indicated by squares and circles respectively, with open symbols indicating healthy individuals and shaded symbols indicating affected individuals. Triangles indicate unaffected individuals with censored gender, and diagonal lines indicate deceased individuals. **b** Sanger sequencing of *STK4* gene confirmed WES findings – a homozygous C to T substitution at position 442 in patients that was heterozygous in both parents. **c** Western blotting of P3-derived EBV-transformed B cell lysate and P1 and P3 fibroblast lysates, demonstrating MST1 deficiency. Control lysates were derived from random healthy individuals. Representative of a minimum of two separate experiments

Laboratory work-up in all three children (Table 1) was remarkable for CD4 lymphopenia, absence of naïve T cells and hypergammaglobulinemia (yet low IgG_2_ and poor pneumococcal responses) without evidence of autoimmunity. Thymic size was normal on computer tomographic imaging (data not shown). Given this clinical picture and a consanguineous background (Fig. 1a), a leaky form of autosomal recessive severe combined immunodeficiency was suspected but known defects were ruled out by a negative genetic screen. Homozygosity mapping was therefore undertaken, followed by WES of two of the three patients. This led to the identification of a novel homozygous nonsense variant (c. 442C > T, p. Arg148Stop) in *STK4*, encoding MST1, that was confirmed to segregate with disease in the family by Sanger sequencing (Fig. 1b). Western blot analysis of patient fibroblast and lymphoblastoid B cell lysates confirmed loss of MST1 protein expression and hence the diagnosis of MST1 deficiency (Fig. 1c).

**Table 1:** Clinical features and laboratory values for patients, demonstrating profound CD4-lymphopenia and reduced lymphoproliferative responses in patients

*Feature*	P1	P2	P3	Normal range
Clinical features				
Eczema & skin infections	+	+	−	
Recurrent sinopulmonary infection	+	+	−	
Oral candidiasis	−	+	−	
Cryptosporidiosis	+	+	+	
Viral infections:				
*Molluscum contagiosum*	+	+	+	
Recurrent VZV	−	−	+	
Severe 1° HSV	+	+	−	
Chronic EBV viremia	+	+	+	
EBV LPD	+	+	−	
Flow cytometric immunophenotyping (*absolute cell counts per* μl *except where* % *indicated*)				
CD3^+^ T cells	815	850	921	1400–8000
CD4^+^ T cells *naive CD4^+^ T cells	2000	26,817	36,818	900–5500
CD8^+^ T cells *naïve CD8^+^ T cells	51,216	59,217	52,328	400–2300
TCR-γδ^+^ (% of T cells)	28	7	15	
HLA-DR^+^ (% of T cells)	84	77	62	
CD16^+^/CD56^+^ NK cells	192	147	298	100–1400
CD19^+^ B cells	439	897	1355	600–3100
CD27^+^IgD^−^ (% of B cells)	2	3	5	
Lymphocyte proliferation assay				
PHA patient (cpm)	11,326	34,343	67,254	
PHA control (cpm)	148,110	122,215	101,164	
SI patient	82	29	94	
SI control	445	407	349	
Immunoglobulins				
*IgG*	18.3	28.8	17.8	3.0–13.3 g/L
*IgA*	2.2	4.47	0.7	0.3–1.29 g/L
*IgM*	0.6	1.13	0.9	0.43–1.9 g/L
*Pneumococcal ab*	<3	17	88	20–200 mg/L
*Tetanus ab*	0.88	1.55	0.49	0.1–10 IU/ml
*Hemophilus B ab*	NT	>9.0	NT	1.0–20 mg/L

*VZV Varicella zoster virus*, *HSV Herpes simplex virus*, *EBV Epstein Barr Virus*, *LPD* lymphoproliferative disease, *PHA* phytohemagglutinin, *cpm* counts per minute, *SI* stimulation index, *ab* specific antibody, *NT* not tested

^a^Laboratory evaluation was performed at 5.5 yrs. (P1), 3.4 yrs. (P2), 1.7 yrs. (P3)

^b^Naïve T cells were defined as CD3 + CD27 + CD45RA+ and as either CD4+ or CD4-

^c^Normal ranges are age matched to P3

Confidence in the disease-causing nature of this variant was increased by the publication of two independent reports linking loss-of-function *STK4* mutations to CID [9, 10]. MST1 deficiency was suggested to cause progressive lymphopenia due to the anti-apoptotic role of the enzyme, but the reduced adhesive and chemotactic potential found in murine Mst1^−/−^ lymphocytes [11, 13, 14, 16] was not fully explored in their human equivalents.

**Fig. 2:**
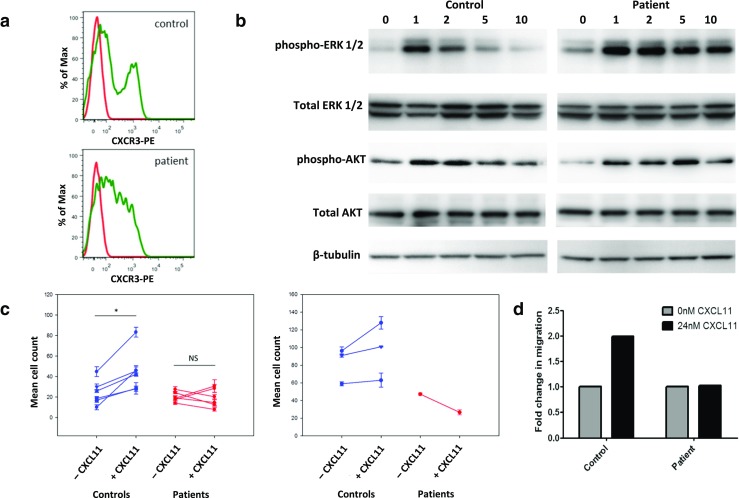
**a** Patient CD4+ T lymphocytes exhibit expression of the CXCL11 receptor CXCR3 (red histogram = unstained control, green histogram = anti-CXCR3-PE antibody) (*n* = 1). **b** EBV-transformed B cells were treated with 12 nM CXCL11 to stimulate CXCR3 before whole cell lysis, Western transfer analysis and probing for phospho-ERK, total ERK, phospho-AKT and total AKT, with β-tubulin as a loading control. Numbers indicate time (mins) of treatment with CXCL11. Representative of three separate experiments. **c** CXCL11 treated patient EBV-transformed B cells (left panel) show reduced binding to ICAM-1-coated chips under flow conditions when compared to control cells (* indicates *P* = 0.0313). Patient PBLs also show reduced binding when compared to control cells (right panel). Each shape represents one of six separate experiments, with 5 replicates for each. Significance was determined using Wilcoxon signed rank tests. **d** CXCL11-treated patient PBLs show unchanged capacity for migration across transwell filters when compared to control cells (*n* = 1)

Prior to the investigation of the adhesive and chemotactic properties of patient peripheral blood leukocytes (PBLs), the chemokine receptor expression of these cells was investigated using flow cytometry. Expression of CXCR3 – a receptor for CXCL11 – was observed in patient CD4^+^ T lymphocytes (Fig. 2a), along with expression of CCR1, CCR2, CCR3, CCR5, CCR6, CCR7, CXCR1, and CXCR5, and slightly reduced expression of CCR9 and CXCR4 (data not shown). Due to the robust expression of CXCR3 by patient cells, the CXCL11-CXCR3 pathway was chosen for subsequent experiments to investigate chemokine responses.

Assessment of ERK1/2 and AKT phosphorylation by Western blot of patient EBV-transformed B-cell lysates demonstrated an intact cellular response to CXCL11 stimulation when compared to control EBV-transformed B-cell lysates (Fig. 2b). These data confirm that chemokine receptor signalling upstream of these proteins is preserved.

The ability of both patient EBV-transformed B cells and patient PBLs to bind to surfaces under flow conditions was investigated using a microfluidic system (Cellix VenaFlux platform). Patient and control cells were exposed to a chip coated with ICAM-1, with or without CXCL11 stimulation, and adhered cells were counted. Control EBV-transformed B cells exhibited a significant positive change in adhesiveness upon stimulation with CXCL11, with the mean cell count increasing from 24.3 to 45.5 (Fig. 2c left panel; *n* = 6, *P* = 0.0313). Conversely, patient EBV-transformed B cells underwent no significant change in adhesiveness in response to the same chemokine, with the mean cell count decreasing from 20.2 to 19.0 (*n* = 6). Similar data were obtained using patient and control primary PBLs, although this experiment could only be carried out once with patient cells due to lack of patient material (right panel). These data suggest that MST1-deficient human lymphocytes are unable to respond to chemokine stimulation by increasing adhesive potential in the same way as control human lymphocytes.

In view of this impaired adhesive response to chemokine, the chemotactic ability of primary patient cells was assessed by performing transwell chemotaxis assays (Fig. 2d). Upon addition of 24 nM CXCL11, control PBLs exhibited a 2 fold-increase in the number of cells in the lower compartment when compared to unstimulated cells. In comparison, migration of patient PBLs was unaltered after the addition of 24 nM CXCL11 (note that this experiment could only be carried out once due to lack of patient material). These data suggest that MST1-deficient patient PBLs are impaired in their chemotactic response to chemokine stimulation. This is consistent with mouse data that demonstrate a role for MST1 in lymphocyte chemotaxis [11].

Together, these observations therefore emphasise the conserved role of MST1 in murine and human lymphocyte adhesion and trafficking [11–14, 17], and offer a novel perspective on the phenotype described in these patients.

## Discussion

CID is a group of immunodeficiencies with diverse genetic etiologies. Recent studies have identified biallelic mutations in *STK4* as a cause of CID. Nehme et al. [10] described two families: the first containing one patient with a homozygous SNP (c.C349T) leading to a premature stop codon (p.Arg117Stop), and the second containing three siblings with a homozygous single base deletion (c.T1103del) resulting in a frameshift and a premature stop codon at position 369 of the MST1 protein. Abdollahpour et al. [9] described three patients (two siblings and their niece) in one family, all with a homozygous SNP (c.G750A) leading to a premature stop codon (p.Trp250Stop). More recently still, a report by Halacli et al. [18] was published detailing a fourth MST1-deficient kindred from Turkey featuring a novel 4 bp deletion in *STK4* (c.58-61delATAG) that results in a premature stop codon.

This study describes a fifth kindred with a different homozygous *STK4* mutation (c. 442C > T, p. Arg148Stop), resulting in absent MST1 protein expression and profound CD4 lymphopenia. The overall clinical picture described here is similar to previous reports, with both sinopulmonary infections and abnormal herpesviral handling including EBV-related lymphoproliferative disease. While autoimmune complications, absent from our patients, were described by Halacli et al. in two patients and Nehme et al. in two of four patients [10], Abdollahpour et al. found no consistent evidence of autoimmunity [9]. The novel finding of cryptosporidiosis in all three affected members of the present kindred is entirely consistent with severely compromised T-cell function.

MST1 deficiency is a primary immunodeficiency that has overlapping but not identical clinical features with other PIDs involving defects in actin cytoskeletal reorganization. MST1 deficiency shares with DOCK8 deficiency and Wiskott-Aldrich Syndrome (WAS) the occurrence of skin abscesses and superficial infections with bacterial (*Staphylococcus aureus*), viral (*Molluscum contagiosum*, *Herpes simplex*, *Varicella zoster* and *Human papilloma virus*) and fungal (*Candida albicans*) pathogens. Furthermore, recurrent respiratory infections and bronchiectasis, as well as T-cell lymphopenia and reduced in vitro T-cell proliferation, are part of all three diseases. Autoimmunity may be a feature of both MST1 deficiency and WAS, in particular autoimmune haemolytic anaemia, thrombocytopenia and neutropenia. Highly elevated serum IgE levels, atopic manifestations such as severe eczema and allergies, and failure to thrive distinguish DOCK8 deficiency and WAS from MST1 deficiency. In addition to these features, marked hypereosinophilia is suggestive of DOCK8 deficiency, whereas microthrombocytopenia in a male is typical for WAS [19, 20]. Thus, when patients exhibit marked CD4 lymphopenia and susceptibility to infections in the absence of a bleeding disorder, MST1 deficiency should be considered along with DOCK8 deficiency [19]. Also on the differential diagnosis for CD4 lymphopenia with EBV lymphoproliferative disease are autosomal recessive abnormalities of T-cell signalling such as ITK and CD27 deficiencies, as well as the X-linked disorder XMEN (MAGT1 deficiency) [21]. Clinically, these disorders and MST1 deficiency may behave very similarly. A thorough diagnostic work-up including molecular genetic testing is advised to inform decision-making around stem cell transplantation, which will often be required.

The cellular phenotype of MST1-deficient human lymphocytes was explored in this study. Patient EBV-transformed B cells and PBLs exhibited reduced LFA-1-dependent adhesiveness, and patient PBLs showed reduced transwell migration after chemokine stimulation, when compared to control cells. Nehme et al. also investigated a potential migratory defect in MST1-deficient patient cells by using transwell assays, and found that chemotaxis in response to CCL19 and CCL21 was impaired, although this was attributed to the observed reduction in expression of CCR7 – consistent with the absence of naïve T cells [10]. In contrast, the present study shows a chemotactic defect despite preserved expression of the relevant chemokine receptor. Moreover, patient cells retained their ability to phosphorylate ERK1/2 and AKT in response to chemokine stimulation, indicating intact or even augmented outside-in signaling. In the mouse, inside-out signalling via Mst1/Rap1/RAPL is vital for the stable arrest of lymphocytes on endothelial surfaces [11] and for immune synapse formation [13]; we therefore speculate that human MST1 performs the same function, which was disrupted in patient cells.

Both Abdollahpour et al. and Nehme et al. investigated the apoptotic potential of lymphocytes from MST1-deficient patients [9, 10]. This was not an area of primary focus within this study, although levels of apoptosis in resting control and patient EBV-transformed B cells were assessed and no consistent difference was found. Recent studies have also suggested a role for MST1 in the regulation of autophagy, although evidence regarding its precise role is conflicting [22–24]. In future, the autophagic potential of primary MST1-deficient human cells should be investigated, as this may contribute to the immune phenotype seen here.

Our data reinforce previous findings in mice that suggest a role for MST1 in LFA-1 activation and polarization [17] and extend these findings to human disease. This reduction in lymphocyte binding potential has ramifications for many immune processes, which likely contributes to the profound phenotype seen in MST1-deficient patients. The relationship between increased apoptosis and decreased lymphocyte adhesion merits further study.
